# Antibiotic prescribing practices in three neonatology units in Kigali, Rwanda. – an observational study

**DOI:** 10.4314/ahs.v20i4.17

**Published:** 2020-12

**Authors:** Peter Thomas Cartledge, Fidel Shofel Ruzibuka, Florent Rutagarama, Samuel Rutare, Tanya Rogo

**Affiliations:** 1 Department of Pediatrics, University Teaching Hospital of Kigali (UTHK), Kigali, Rwanda; 2 Department of Pediatrics, Yale University (USA), Rwanda Human Resources for Health (HRH) Program, Kigali, Rwanda; 3 Department of Pediatrics, University of Rwanda, Kigali, Rwanda; 4 Department of Pediatrics, Rwanda Military Hospital, Kigali, Rwanda; 5 Department of Pediatrics, BronxCare Health System, Bronx, NY, USA

**Keywords:** (MeSH): Antimicrobial stewardship, anti-bacterial agents, neonatal sepsis, sepsis, infant mortality, neonatal intensive care units, Africa, Rwanda

## Abstract

**Introduction:**

There is limited published data on antibiotic use in neonatal units in resource-poor settings.

**Objectives:**

This study sought to describe antibiotic prescribing practices in three neonatology units in Kigali, Rwanda.

**Methods:**

A multi-center, cross-sectional study conducted in two tertiary and one urban district hospital in Kigali, Rwanda. Participants were neonates admitted in neonatology who received a course of antibiotics during their admission. Data collected included risk factors for neonatal sepsis, clinical signs, symptoms, investigations for neonatal sepsis, antibiotics prescribed, and the number of deaths in the included cohort.

**Results:**

126 neonates were enrolled with 42 from each site. Prematurity (38%) followed by membrane rupture more than 18 hours (25%) were the main risk factors for neonatal sepsis. Ampicillin and Gentamicin (85%) were the most commonly used first-line antibiotics for suspected neonatal sepsis. Most neonates (87%) did not receive a second-line antibiotic. Cefotaxime (11%), was the most commonly used second-line antibiotic. The median duration of antibiotic use was four days in all surviving neonates (m=113). In neonates with negative blood culture and normal C-reactive protein (CRP), the median duration of antibiotics was 3.5 days; and for neonates, with positive blood cultures, the median duration was 11 days. Thirteen infants died (10%) at all three sites, with no significant difference between the sites.

**Conclusion:**

The median antibiotic duration for neonates with normal lab results exceeded the recommended duration mandated by the national neonatal protocol. We recommend the development of antibiotic stewardship programs in neonatal units in Rwanda to prevent the adverse effects which may be caused by inappropriate or excessive use of antibiotics.

## Introduction

Infection is responsible for approximated 35% of all neonatal deaths 1. Neonatal mortality in Rwanda is 20 per 1000 live births, and neonatal infection is the third leading cause of neonatal mortality after prematurity and birth asphyxia 2. Antibiotics are the most commonly prescribed medications in neonatal units 3,4. When used appropriately, antibiotics save the lives of neonates with bacterial infections. However, inappropriate or excessive use of antibiotics in neonates leads to adverse outcomes such as the increased risk of late-onset sepsis (LOS), necrotizing enterocolitis (NEC), the emergence of multidrug-resistant organisms (MDROs), and increased rate of invasive candidiasis 4,5. Giving antibiotics when they are not required also adds to the cost of care, increases the workload on nursing and clinical staff and has the potential to extend the length of hospital stay.

In resource-rich settings, antibiotic stewardship is recognized as a critical patient safety and quality initiative to combat unnecessary antibiotic use to optimize clinical outcome while minimizing the harmful consequences of antimicrobial use^6^. A comprehensive approach to antibiotic stewardship includes accurate measurement of antibiotic use, improved diagnostic techniques, rational selection of empiric therapy, and continual re-evaluation and de-escalation or discontinuation when appropriate 4. Hoever, there is limited published data on antibiotic use in resource-poor settings 7.

The Rwanda Neonatal protocol advocates for the firstline empirical treatment of neonatal sepsis with Ampicillin and Gentamicin 8. Empirical antibiotics should be discontinued after 48 hours in neonates with a stable clinical condition and normal laboratory results, while infants with clinical sepsis and a positive C-reactive protein (CRP) should receive seven days of antibiotics and clinical meningitis for a minimum of 14 days.

## Study objectives

This study sought to describe antibiotic prescribing practices (antibiotic used and duration) in three Neonatology Units in Kigali, Rwanda. Secondary objectives were to describe the all-cause mortality, frequencies of risk factors for sepsis, and clinical features of sepsis in the recruited patients.

## Methodology

**Study design:** A multi-center, cross-sectional study. Reporting of this study has been verified in accordance with the STROBE (Strengthening the Reporting of Observational Studies in Epidemiology) checklist 9.

**Study sites:** The study was conducted in three hospitals in Kigali, namely; University Teaching Hospital of Kigali (UTHK), Rwanda Military Hospital (RMH) and Kibagabaga District Hospital (KDH) 10. UTHK and RMH are tertiary teaching sites, while KDH is an urban district hospital. All three sites have maternity units.

**Participants:** Inclusion criteria were neonates admitted in neonatology units at UTHK, RMH, and KDH who were given a course of antibiotics during their admission to the neonatal unit (i.e. neonates admitted directly from maternity units). Neonates who were admitted to other units and/or from outside hospitals, i.e. neonates from the community, were excluded. Subjects were opportunistically recruited by the principal investigator (PI) between August to December 2017. The Principle Investigator (PI), was a pediatric resident, based in Kigali, who would intermittently visit the study sites, and recruit patients available at that time point. The PI then followed-up these patients, collecting the necessary data during the admission. No data on non-recruitment was taken.

**Outcomes:** The primary objective of the study was to describe antibiotic prescribing practices, predefined as antibiotic used and duration of antibiotic use. The duration was described in days of antibiotic used. To take into account for the amount of time neonates spent as inpatients, a second descriptor for antibiotic duration was used, namely the Antibiotic-Use-Ratio (AUR). AUR was pre- defined as the number of days an infant was exposed to one or more antimicrobial agents divided by the total length of hospital stay 11. The secondary outcome was to describe the all-cause mortality, frequencies of risk factors for sepsis (as defined by Rwanda Ministry of Health 8, and clinical features of sepsis in the recruited patients

**Data-collection:** A data-collection tool was designed specifically for use in this study and was reviewed at the University of Rwanda (UR) pediatric academic team for content validity. This data-collection tool was piloted on the case-files of five neonates to ensure the feasibility of use. Data were collected prospectively from patient files (note-review) onto a paper version of the data-collection tool, recruited between 2^nd^ August and 22^nd^ December 2017. Ethical clearance was gained to question parents if any data was missing from the patient file, however this was not required.

**Data management:** Data were then entered into Epi-data before analysis using Statistical Package for the Social Sciences (SPSS) version 24.

**Sample size:**

Goal sample size was 42 neonates from each site powered to identify a difference of 2 days between district and tertiary hospital duration of antibiotic use, with 95% confidence (2-sided) with 80% power and a ratio 2:1 for tertiary:district hospital sites.

**Statistical analysis:** For categorical data, ordinal outcomes were analyzed with Chi-square. For cells smaller than five, Fischer's exact test was employed. For continuous data, means were compared with Student's t-test or ANOVA dependent on group size. Non-normally distributed data were described using medians and compared using the Mann-Whitney non-parametric test.

## Results

**Characteristics of recruited participants:** One hundred and twenty-six neonates were enrolled with 42 neonates from each study site (Table 1). The neonates had a mean gestational age of 36 weeks, and the majority were male (62%). There were more preterm neonates at UTHK (p=0.03). Fifty-four percent of neonates were born by Caesarean-section (CS). The median length of hospital stay was five days, with no significant difference between the three hospital sites.

**Table 1:** Characteristics of recruited participants

	KDH (n=42)	UTHK (n=42)	RMH (n=42)	Total (n=126)	p-value
**Gender (male)**	30 (71.4%)	20 (47.6%)	28 (66.7%)	78 (61.9%)	p=0.58 (df=2)*χ*
**Mean** **gestation (weeks)**	37.2 (±3.3)	35.9 (±3.5)	36.3 (±4.4)	36.4 (±3.7)	p=0.290 (df=2)*α*
**Gestational groups** Term (>37 weeks) 32–37 weeks 28–32 weeks <28 weeks Unknown	24 (57.1%) 7 (16.7%) 1 (2.4%) 1 (2.4%) 9 (21.4%)	17 (40.5%) 20 (47.6%) 4 (9.5%) 0 (0%) 1 (2.4%)	18(42.9%) 9 (21.4%) 1 (2.4%) 2 (4.8%) 12 (28.6%)	59(46.8%) 36 (28.6%) 6 (4.8%) 3 (2.4%) 22 (17.5%)	p=0.03 (df=8)*χ*
**Mean birth weight** **(Kg)**	2.64 (±0.71)	2.42 (±0.85)	2.57 (±0.91)	2.54 (±0.83)	p=0.482 (df=2)*α*
**Mode of delivery:** Vaginal Elective LSCS Emergency LSCS Data not available	26 (61.9%) 4 (9.5%) 11 (26.2%) 1 (2.4%)	13 (31.0%) 6(14.3%) 22 (52.4%) 1 (2.4%)	17 (40.5%) 6 (14.3%) 19 (45.2%) 0 (0.0%)	56 (44.4%) 16 (12.7%) 52 (41.3%) 2 (1.6%)	p=0.125 (df=6)*χ*
**Place of birth** Home On the way to HF District Hospital Health Centre Tertiary Hospital Data not known	0 (0.0%) 1 (2.4%) 34 (81.0%) 6 (14.3%) 0 (0.0%) 1 (2.4%)	0 (0.0%) 0 (0.0%) 1 (2.4%) 0 (0.0%) 39 (92.9%) 2 (4.8%)	1 (2.4%) 0 (0.0%) 8 (19.0%) 2 (4.8%) 30 (71.4%) 1 (2.4%)	1 (0.8%) 1 (0.8%) 43 (34.1%) 8 (6.3%) 69 (54.8%) 4 (3.2%)	p<0.001 (df=10)*χ*
**Mean LOS** All neonates Surviving neonates Neonates who died	5.4 days (±3.8) 5.8 days (±3.8) 2.5 days (±2.4)	7.5 days (±7.6) 7.9 days (±7.6) 0.5 days (±0.7)	7.7 days (±7.2) 7.7 days (±6.9) 8.0 days (±10.9)	6.9 days (±6.5) 7.2 days (±6.4) 4.0 days (±6.6)	p=0.174 p=0.311 p=0.348*α*
**Median LOS** All neonates Surviving neonates Neonates who died	4 days 5 days 2.5 days	5 days 5 days 0.5 days	5.5 days 5.5 days 3.5 days	5 days 5 days 1.5 days	p=0.498 p=0.725 p=0.321*M*
**Admission** **diagnosis of** **septicemia**	40 (95.2%)	38 (90.5%)	42 (100%)	120 (95.2%)	p=0.122*χ*
**Mortality rate**	6 (14.3%)	2 (4.8%)	5 (13.5%)	13 (10.3%)	p=0.331*r*
**Sepsis as cause of** **death**	4/6 (66.7%)	1/2 (50.0%)	5/5 (100%)	10/13 (76.9%)	p=0.244*r*

LOS=Length of stay; LSCS=Caesarean section; UTHK=University Teaching Hospital of Kigali; RMH=Rwanda Military Hospital; KDH=Kibagabaga District Hospital; HF=health facility; LOS=length of stay ±SD = *standard deviation*

χ *Pearson Chi-Squared*

r *Fischer's exact test*

α *ANOVA*

M Non-parametric – Mann-Whitney test

Duration of antibiotic use: The distribution of duration of antibiotic use was not normally distributed. Therefore, the median was used to describe the duration of antibiotic use with the statistical comparison between sites being undertaken with non-parametric tests (Mann-Whitney test). There were significantly different prescribing habits between the hospital sites regarding the duration of antibiotic use and in the antibiotic to the length of stay ratio (ALR) (Table 2). Surviving neonates who had a negative blood culture (n=64) received a median of four days of antibiotics (Table 3). Neonates with both a normal CRP and blood culture received a shorter course of antibiotics of 3.5 days. There was a significantly higher median duration of antibiotic use for neonates who had late neonatal sepsis compared to early sepsis (p=0.040).

**Table 2:** Length and choice of antibiotics

	KDH (n=42)	UTHK (n=42)	RMH (n=42)	Total (n=126)	p-values
**Median length of antibiotic use** **(days)** All neonates (n=126) Surviving neonates (113)	4 (±6.0) 4.5 (±6.3)	3 (±2.6) 3 (±2.6)	5 (±8.5) 5 (±8.3)	4 (±6.3) 4 (±6.3).	p=0.080*M* p=0.011*M*
**Median AUR** All neonates Surviving neonates	0.83 (±0.4) 0.83 (±0.4)	0.50 (±0.3) 0.50 (±0.3)	0.88 (±0.7) 0.88 (±0.7)	0.8 (±0.5) 0.8 (±0.5)	p<0.001*M* p<0.001*M*
**Median length of antibiotics** **(days)** Blood culture positive Blood culture negative	NA*	11 (n=1) 3 (±2.8)	12.5 (±7.9, n=10) 5 (±8.5)	11 (±8.2). 4 (±6.0).	p=0.182*M* p=0.141*M*
**Median AUR** Blood culture positive Blood culture negative	NA*	0.143 (n=1) 0.5 (±0.3)	0.92 (±0.3) 0.88 (±0.79)	0.88 (±0.38) 0.67 (±0.6)	p=0.182*M* p=0.005*M*
**First line antibiotics used** Ampicillin and Gentamicin Ampicillin and Cefotaxime Cefotaxime alone Meropenem and vancomycin	42 (100%) 0 (0.0%) 0 (0.0%) 0 (0.0%)	40 (95.2%) 0 (0.0%) 0 (0.0%) 2 (4.8%)	25 (59.5%) 15 (35.7%) 2 (4.8%) 0 (0.0%)	107 (84.9%) 15 (11.9%) 2 (1.6%) 2 (1.6%)	p=0.010*r*
**Second line antibiotics used** No second line abx Cefotaxime Cloxacillin Vancomycin Imipenem/Meropenem Ciprofloxacin Metronidazole	34 (81%) 8 (19%) 0 (0.0%) 0 (0.0%) 0 (0%) 0 (0%) 0 (0%)	39 (92.9%) 2 (4.8%) 0 (0.0%) 1 (2.4%) 0 (0%) 0 (0%) 0 (0%)	37 (88.1%) 4 (9.5%) 1 (2.4%) 0 (0.0%) 0 (0%) 0 (0%) 0 (0%)	110 (87.3%) 14 (11.1%) 1 (0.8%) 1 (0.8%) 0 (0%) 0 (0%) 0 (0%)	p=0.112*r*

UTHK=University Teaching Hospital of Kigali; RMH=Rwanda Military Hospital; KDH=Kibagabaga District Hospital; AUR=Antibiotic Use Ratio; ± standard deviation

M Mann-Whitney U test

r Fischer's exact test

(n=) figure is given this reflects datasets where sufficient data was available from the case-file for this outcome

* No blood culture facilities at KDH

**Table 3:** Length of antibiotic use in surviving neonates

	Median length of antibiotic use (Days)	*M*p-value	AUR	*M*p-value
**All surviving neonates** (n=113)	4 (±6.3)	-	0.8 (±0.5)	-
**Blood culture** Positive (n=8) Negative (n=64)	12.5 (±7.2) 4.0 (±6.1)	p=0.007	0.88 (±0.4) 0.67 (±0.6)	p=0.169
**CRP** Positive (n=20) Negative (n=78)	8.5 (±11.1). 3.0 (±3.1)	p<0.001	0.97 (±0.9) 0.75 (±0.3)	p=0.003
**FBC** Abnormal (n=15) Normal	7.0 (±7.3). 4.0 (±6.0)	p=0.007	0.88 (±0.3) 0.75 (±0.5)	p=0.276
**Blood culture and CRP combined** Either abnormal (n=10) Both normal (n=52)	5.0 (±13.6). 3.5 (±4.5)	p=0.076	0.96 (±1.2) 0.67 (±0.3)	p=0.015
**Diagnosis of sepsis** Early sepsis (n=113) Late sepsis (n=13)	4.0 (±4.9) 9.0 (±12.5)	p=0.040	0.80 (±0.4) 0.96 (±1.1).	p=0.032

AUR=Antibiotic Use Ratio

M Mann-Whitney U test

Choice of antibiotic: Ampicillin and Gentamicin (84.9%) were the most commonly used first-line antibiotics for suspected neonatal sepsis (Table 2). Most neonates (87%) did not receive second-line antibiotics. Cefotaxime (11%), was the most commonly used second-line antibiotic.

Mortality in recruited patients: [A1]Overall mortality, in recruited patients, at all three sites was 10% with no significant difference between the sites. Sepsis was the cause of death in 77% of deaths (Table 1).

Causative organisms: Two of the three sites (UTHK and RMH) have microbiology laboratory facilities and therefore, can perform blood cultures. Seventy-eight (93%) neonates had a blood culture performed in the sites with these facilities. Of these, 11 (14%) had a positive blood culture (seven gram-negative and four gram-positive). The following organisms were reported: *Klebsiella* spp (n=1); *Staphylococcus aureus* (n=2); Coagulase-negative *staphylococcus* (n=1); *E. coli* (n=3); Non-specific “Gram-negative bacilli” (n=3); Non-specific “Gram-positive cocci” (n=1).

Frequencies of risk factors, and clinical features of sepsis[A2]: Prematurity was the most common risk factor for sepsis (38%) followed by prolonged rupture of membranes (25%) (Table 4). Neonates each had a mean of 1.2 risk factors, with only 34% having two or more risk factors documented. The most frequent clinical feature of sepsis was difficulty in feeding (34%) (Table 5).

**Table 4:** Length and choice of antibiotics

	KDH (n=42)	UTHK (n=42)	RMH (n=42)	Total (n=126)	p-values
**Median length of antibiotic use** **(days)** All neonates Surviving neonates	4 (±6.0) 4.5 (±6.3)	3 (±2.6) 3 (±2.6)	5 (±8.5) 5 (±8.3)	4 (±6.3) 4 (±6.3).	p=0.080 p=0.011
**Median AUR** All neonates Surviving neonates	0.83 ( 0.83 (	0.50 ( 0.50 (	0.88 ( 0.88 (	0.8 ( 0.8 (	p<0.001 p<0.001
**Median length of antibiotics** **(days)** Blood culture positive Blood culture negative	NA*	11 (n=1) 3 (±2.8)	12.5 (±7.9, n=10) 5 (±8.5)	11 (±8.2). 4 (±6.0).	p=0.182 p=0.141
**Median AUR** Blood culture positive Blood culture negative	NA*	0.143 (n=1) 0.5 (±0.3)	0.92 (±0.3) 0.88 (±0.79)	0.88 (±0.38) 0.67 (±0.6)	p=0.182 p=0.005
**First line antibiotics used** Ampicillin and Gentamicin Ampicillin and Cefotaxime Cefotaxime alone Meropenem and vancomycin	42 (100%) 0 (0.0%) 0 (0.0%) 0 (0.0%)	40 (95.2%) 0 (0.0%) 0 (0.0%) 2 (4.8%)	25 (59.5%) 15 (35.7%) 2 (4.8%) 0 (0.0%)	107 (84.9%) 15 (11.9%) 2 (1.6%) 2 (1.6%)	p=0.010
**Second line antibiotics used** No second line abx Cefotaxime Cloxacillin Vancomycin Imipenem/Meropenem Ciprofloxacin Metronidazole	34 (81%) 8 (19%) 0 (0.0%) 0 (0.0%) 0 (0%) 0 (0%) 0 (0%)	39 (92.9%) 2 (4.8%) 0 (0.0%) 1 (2.4%) 0 (0%) 0 (0%) 0 (0%)	37 (88.1%) 4 (9.5%) 1 (2.4%) 0 (0.0%) 0 (0%) 0 (0%) 0 (0%)	110 (87.3%) 14 (11.1%) 1 (0.8%) 1 (0.8%) 0 (0%) 0 (0%) 0 (0%)	p=0.112

AUR=Antibiotic Use Ratio; ± standard deviation

M Mann-Whitney U test

r Fischer's exact test

(n=) figure is given this reflects datasets where sufficient data was available from the case-file for this outcome

* No blood culture facilities at KDH

**Table 5:** Clinical features of sepsis

	Total (n=126)
Difficulty in feeding	43 (34.1%)
Abnormal temperature (Fever OR hypothermia)	30 (23.8%)
Tachypnoea	26 (20.6%)
Hypoxia	22 (17.5%)
Convulsions	14 (11.1%0
Reduced movements	6 (4.8%)
Tachycardia	3 (2.4%)
Delayed capillary refill time	0 (0.0%)
**Mean number of features in each neonate**	**1.14 (±1.08)**

± standard deviation

## Discussion

This study aimed to describe antibiotic prescribing practices in three Neonatology Units in Kigali, Rwanda. We found that the median duration of antibiotics was four days in surviving neonates with 85% receiving ampicillin and gentamicin as first-line antibiotics. The median duration of antibiotics is consistent with other reported studies 12. The most common risk factor for sepsis was prematurity, and the most common clinical sign of sepsis was difficulty in feeding, both of which are well known in neonatal sepsis 13.

The mean number of risk factors for each neonate was only 1.2, which might suggest that antibiotics were being used too frequently when significant risks were not present. However, this is speculative as this study did not include infants who did not receive antibiotics, and some risk factors may not have been thoroughly documented in the case-files. The evaluation of asymptomatic neonates for sepsis is controversial, but several studies have shown that application of risk stratification can result in decreases in empiric antibiotic use 14–16. Duration of antibiotics was analyzed for surviving neonates as those neonates that died may have done so before completing a course of antibiotics. Surviving neonates with negative blood cultures received a median of four days of antibiotics. There was a significant variation in median antibiotic duration between study sites (p=0.011), varying between three and five days at CHUK and RMH respectively. This finding was despite a national protocol for antibiotic use that recommends discontinuation of antibiotics after 48 hours in well infants with normal laboratory results. Furthermore, there was a non-significantly higher number of deaths in the recruited patients at the unit using longer courses of antibiotics (Table 1). A wide variation on antibiotic use is not unusual - a retrospective cohort study of neonates in 127 NICUs across the U.S. state of California reported a forty-fold variation in NICU antibiotic prescribing practice, despite similar clinical burdens^17^. Neonatal units in resource-limited settings should monitor antibiotic use to reduce unnecessary use and reduce adverse effects. Implementation of antibiotic stewardship has been shown to decrease antibiotic use and decrease antimicrobial resistance 18.

The most frequently used combination of empirical antibiotics was ampicillin and gentamicin, reflecting the recommendations of the Rwandan national protocol. However, a 2016 study of 128 positive neonatal blood cultures at UTHK in Rwanda, found zero percent sensitivity to ampicillin with 13% sensitivity to gentamicin^2^. Also, 13% of infants in this study received cefotaxime as a first-line antibiotic. First-line cephalosporin use in neonatology units is known to increase the risk of resistance and should be preserved for empirical treatment of neonatal meningitis 19,20. Antibiotic resistance is estimated to be responsible for 30% of deaths from neonatal sepsis, particularly in low and middle-income countries 21.

One of the three hospitals in this study did not have a microbiology laboratory. However, even with the lack of access to blood culture, this hospital (KDH) gave shorter courses of antibiotics. Concerns about the reliability of blood cultures (mainly if the volume of blood sampled is small or the mother received intrapartum antibiotics) can lead to prolongation of antibiotic treatment. Also, delay in reporting negative blood culture results causes clinicians to continue antibiotic treatment while the results are pending. Automated blood culture systems have been shown to identify >94% of bacteria within 48 hours of incubation 22,23. As these hospitals acquire automated systems in their laboratories, duration of therapy should be reassessed to see if it shortens with greater culture reliability.

Neonates received a median of four days, three days, and 3.5 days despite a negative blood culture, CRP, or both (Table 5). Antibiotics should be stopped after 48 hours if the blood culture is negative; however, clinicians should consider clinical signs and laboratory results to decide on the duration of antimicrobial therapy^24^–^26^.

**Table 5:** Length of antibiotic use in surviving neonates

	Median length of antibiotic use (Days)	*M*	AUR	*M*
**All surviving neonates**	4 (±6.3)	-	0.8 (±0.5)	-
**Blood culture** Positive (n=8) Negative (n=64)	12.5 (±7.2) 4.0 (±6.1)	p=0.007	0.88 (±0.4) 0.67 (±0.6)	p=0.169
**CRP** Positive (n=20) Negative (n=78)	8.5 (±11.1). 3.0 (±3.1)	p<0.001	0.97 (±0.9) 0.75 (±0.3)	p=0.003
**FBC** Abnormal (n=15) Normal	7.0 (±7.3). 4.0 (±6.0)	p=0.007	0.88 (±0.3) 0.75 (±0.5)	p=0.276
**Blood culture and CRP combined** Either abnormal (n=10) Both normal (n=52)	5.0 (±13.6). 3.5 (±4.5)	p=0.076	0.96 (±1.2) 0.67 (±0.3)	p=0.015
**Diagnosis of sepsis** Early sepsis (n=113) Late sepsis (n=13)	4.0 (±4.9) 9.0 (±12.5)	p=0.040	0.80 (±0.4) 0.96 (±1.1).	p=0.032

AUR=Antibiotic Use Ratio

M Mann-Whitney U test

## Limitation

This study had a number of limitations. Data was collected from the patient records. Therefore the data was reliant on the completness and accuracy of the patient files. CRP results were only reported as positive or negative, without specifying the quantitative values for a positive CRP. This could impact the duration of antibiotics used as antibiotics can be discontinued if the positive CRP value is low or trending down. In addition, this study is not powered to assess if antibiotic choice and duration have an impact on mortality.

## Clinical implications of the results and recommendations

Governmental bodies, hospitals, and individual clinicians should do more to standardize antibiotic use to avoid site-dependent variation in prescribing. Antibiotic resistance is a public health emergency and, currently, it is an under-researched area 27, and more research is needed to identify effective country-specific and even site-specific neonatal antibiotic protocols for neonatal sepsis 28. We also recommend the initiation of antibiotic stewardship programs in neonatal units in Rwanda to reduce the adverse effects which may be caused by the inappropriate or excessive use of antibiotics in neonates.

## Conclusion

The median antibiotic duration for neonates with normal lab results exceeded the recommended duration by the national neonatal protocol. Based on this finding, we recommend the development of antibiotic stewardship programs, in neonatal units, in Rwanda to prevent the adverse effects which may be caused by inappropriate or unneccesarily prolonged use of antibiotics.
